# Randomized Clinical Trials in Cerebrovascular Neurosurgery From 2018 to 2022

**DOI:** 10.7759/cureus.52397

**Published:** 2024-01-16

**Authors:** Ari D Kappel, Hillary B Nguyen, Kai U Frerichs, Nirav J Patel, Mohammad A Aziz-Sultan, Rose Du

**Affiliations:** 1 Neurosurgery, Brigham and Women's Hospital, Boston, USA

**Keywords:** aneurysm, hemorrhage, stroke, randomized controlled trials, neurosurgery, cerebrovascular

## Abstract

There has been an exponential increase in randomized controlled trials (RCTs) on cerebrovascular disease within neurosurgery. The goal of this study was to review, outline the scope, and summarize all phase 2b and phase 3 RCTs impacting cerebrovascular neurosurgery practice since 2018.

We searched PubMed, MEDLINE, Embase, ClinicalTrials.gov, and the Cochrane Central Register of Controlled Trials (CENTRAL) databases for relevant RCTs published between January 1, 2018, and July 1, 2022. We searched for studies related to eight major cerebrovascular disorders relevant to neurosurgery, including acute ischemic stroke, cerebral aneurysms and subarachnoid hemorrhage, intracerebral hemorrhage, subdural hematomas, cerebral venous thrombosis, arteriovenous malformations, Moyamoya disease and extracranial-intracranial bypass, and carotid and intracranial atherosclerosis. We limited our search to phase 2b or 3 RCTs related to cerebrovascular disorders published during the study period. The titles and abstracts of all relevant studies meeting our search criteria were included. Pediatric studies, stroke studies related to rehabilitation or cardiovascular disease, study protocols without published results, prospective cohort studies, registry studies, cluster randomized trials, and nonrandomized pivotal trials were excluded.

From an initial total of 2,797 records retrieved from the database searches, 1,641 records were screened after duplicates and studies outside of our time period were removed. After screening, 511 available reports within our time period of interest were assessed for eligibility. Pediatric studies, stroke studies related to rehabilitation or cardiovascular disease, study protocols without published results, prospective cohort studies, registry studies, cluster randomized trials, and nonrandomized pivotal trials were excluded. We found 80 unique phase 2b or 3 RCTs that fit our criteria, with 165 topic-relevant articles published within the study period.

Numerous RCTs in cerebrovascular neurosurgery have been published since 2018. Ischemic stroke, including mechanical thrombectomy and thrombolysis, accounted for a majority of publications, but there were large trials in intracerebral hemorrhage, subdural hemorrhage, aneurysms, subarachnoid hemorrhage, and cerebral venous thrombosis, among others. This review helps define the scope of the large RCTs published in the last four years to guide future research and clinical care.

## Introduction and background

Earlier research conducted by our team comprehensively reviewed randomized controlled trials (RCTs) in cerebrovascular neurosurgery published between 2016 and 2017 [1]. At the time, studies covered management for ischemic stroke, aneurysms, subarachnoid hemorrhage, intracerebral hemorrhage, and carotid stenosis. Many important RCTs in cerebrovascular neurosurgery have been published since. These studies further explore questions surrounding indications and timing of mechanical thrombectomy for acute ischemic stroke, as well as new devices and additional strategies for acute management. Controversies regarding clipping versus coiling and endovascular versus surgical intervention for intracranial aneurysms continue, with the ongoing development of new technology. Sequelae of subarachnoid hemorrhage, such as delayed cerebral ischemia and vasospasm, also remain significant clinical challenges. Busy clinicians may benefit from a study outlining the scope of the newest RCTs published in the field and a brief summary of major trials affecting clinical practice. The goal of this study was to review, outline, and summarize all of the high-quality phase 2b and phase 3 RCTs impacting cerebrovascular neurosurgery practice between 2018 and 2022.

## Review

Materials and methods

We searched PubMed, MEDLINE, Embase, ClinicalTrials.gov, and the Cochrane Central Register of Controlled Trials (CENTRAL) databases for relevant RCTs published between January 1, 2018, and July 1, 2022 (last accessed July 1, 2022). We searched for studies related to cerebrovascular neurosurgery, including eight key cerebrovascular disorders: acute ischemic stroke, cerebral aneurysms, subarachnoid hemorrhage, intracerebral hemorrhage, subdural hematomas, cerebral venous thrombosis (CVT), arteriovenous malformations, Moyamoya disease and extracranial-intracranial bypass, and carotid and intracranial atherosclerosis. We searched for (("Clinical Trial" AND ("Phase 2b" OR "Phase IIb" OR "Phase III" OR “Phase 3”)) AND (brain OR cereb* OR neuro*) with specific terms for each cerebrovascular disorder of interest (stroke OR ischaemi* OR ischemi* OR infarct*)); (aneurysm* OR subarachnoid OR hemorrhage OR haemorrhage OR SAH)); (hemorrhag* OR haemorrhag* OR ICH OR IPH OR intracranial OR intraparenchymal)); (subdural OR hemorrhage OR haemorrhage OR hematoma OR haematoma OR SDH)); (“venous thrombosis” OR vein OR thrombosis OR venous OR CVST OR CSVT OR CVT)); ( AVM OR Arteriovenous OR "arteriovenous malformation"); (moya OR moyamoya OR arteriopathy OR bypass); (atherosclerosis OR “carotid stenosis” OR “carotid artery stenosis” OR endarterectomy OR CEA OR CAS)). The titles and abstracts for relevant studies were reviewed, and all phase 3 and phase 2b RCTs were included. Pediatric studies, stroke studies related to rehabilitation or cardiovascular disease, study protocols without published results, prospective cohort studies, registry studies, cluster randomized trials, and nonrandomized pivotal trials were excluded. 

Results

**Figure 1:**
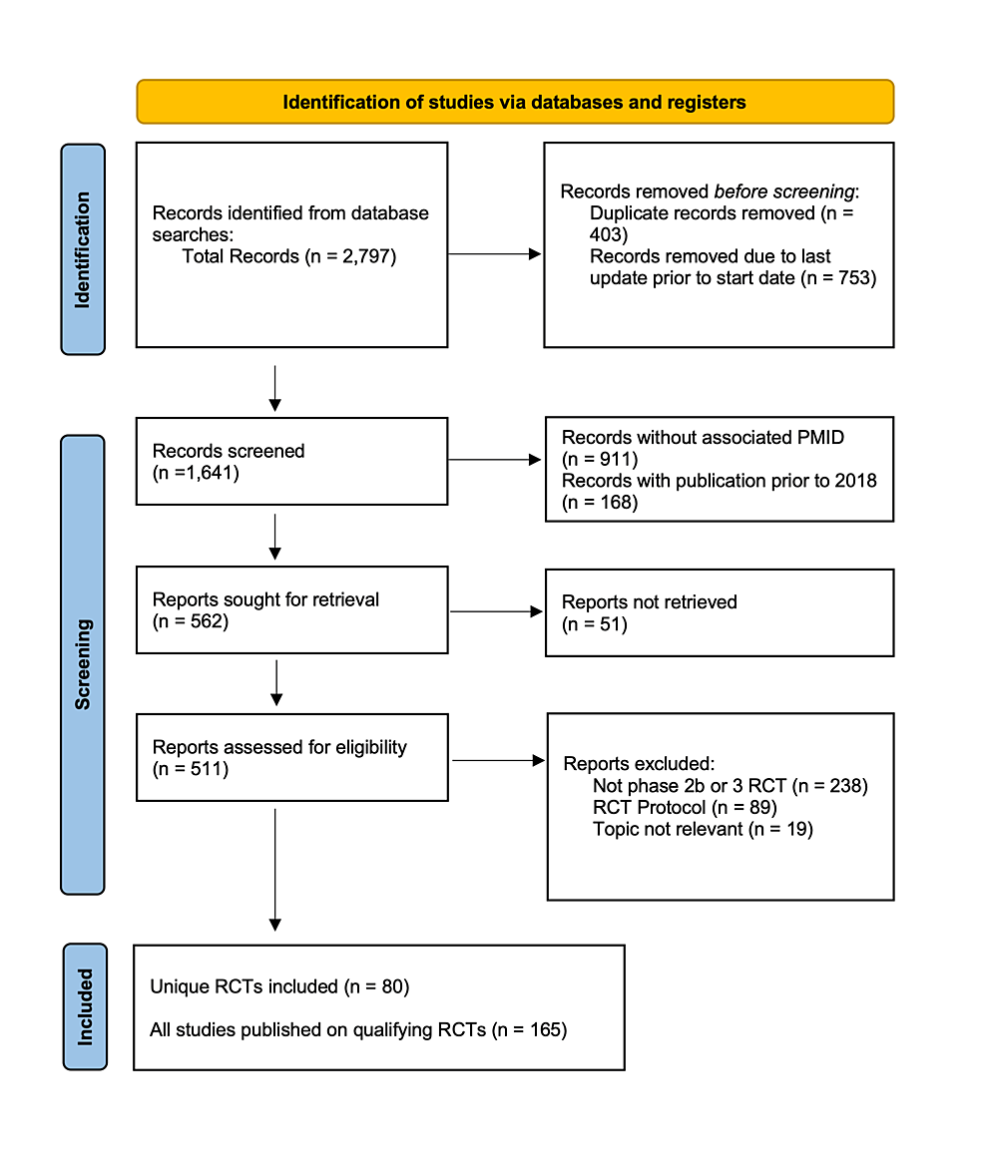
PRISMA flowchart details the results of the literature search for all randomized controlled trials (RCTs) in cerebrovascular neurosurgery published between January 1, 2018, and July 1, 2022. PRISMA, Preferred Reporting Items for Systematic Reviews and Meta-Analyses

Using the aforementioned search strategy, 2,797 records were retrieved from five online databases (last accessed July 1, 2022). We removed 403 duplicates and 753 records with the last updates listed as before January 1, 2018, on ClinicalTrials.gov. A PubMed search of the clinical trial numbers of all remaining 1,641 records returned 730 results. An additional 168 records with publications before 2018 were removed, and 511 were retrieved for further screening of their titles and abstracts. The 238 records that were not phase 2b or 3 RCTs were excluded. Eighty-nine records were protocols for RCTs, and 19 records were not relevant to the topic, with the majority being related to stroke rehabilitation or cardiac interventions for stroke. Eighty RCTs fit our criteria, with 165 articles based on these trials published within the study period (Figure 1).

**Figure 2:**
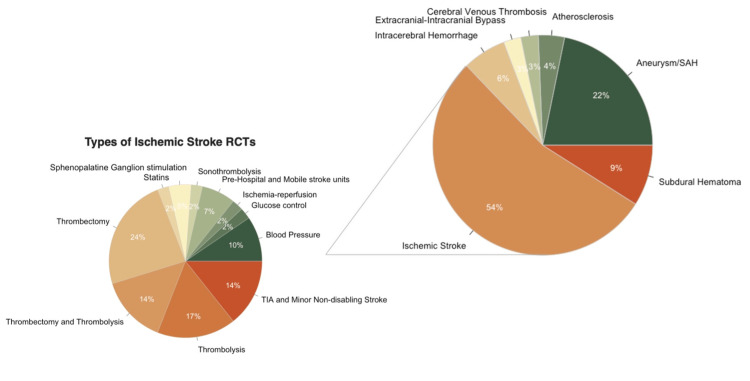
Breakdown of randomized controlled trials (RCTs) included in the review. Eight phase 2b or 3 RCTs with 165 articles published within the study period were included. The majority of trials reviewed (44/80, 54%) were in ischemic stroke, while aneurysm or subarachnoid hemorrhage trials were the second most common (17/80, 22%), with fewer trials in other categories.

Of the 80 trials included in our study, 63 were registered with ClinicalTrials.gov (National Clinical Trial [NCT]), 6 were registered with the International Standard Randomized Controlled Trial Number (ISRCTN) registry, 4 were registered on the Japanese University Hospital Medical Information Network (UMIN) Clinical Trials Registry, 3 were registered with the Chinese Clinical Trials Registry (ChiCTR), 1 was registered with the Netherlands Trial Register (NL), 1 was registered on the German Deutsches Register Klinischer Studien (DRKS) Clinical Trials Registry, 1 was registered on the Australian New Zealand Clinical Trials Registry (ACTRN), and 1 was not registered. The majority of trials reviewed (44/80, 54%) were in ischemic stroke, while aneurysm or subarachnoid hemorrhage trials were the second most common (17/80, 22%), with fewer trials in other categories (Figure 2).

Ischemic stroke/mechanical thrombectomy

Timing and Indications

Strong data from previously published RCTs support mechanical thrombectomy (MT) for acute ischemic stroke (AIS) due to large vessel occlusion (LVO), including MR CLEAN [2], EXTEND-IA [3], ESCAPE [4], REVASCAT [5], SWIFT PRIME [6], and THRACE [7]. DAWN [8] and DEFUSE-3 [9] extend the time window for intra-arterial thrombectomy (IAT) to 24 hours and reinforce the role of perfusion imaging in selecting patients for MT. In DAWN, IAT plus standard medical care 6 to 24 hours after LVO was superior to medical management alone [8]. In DEFUSE-3 [9], thrombectomy plus medical therapy 6 to 16 hours after the last-known well (LKW) lowered 90-day modified Rankin scale (mRS) and mortality rates [9]. The Brazilian RESILIENT [10] trial showed that IAT for LVO was feasible, safe, and effective at a large scale in a low-resource public healthcare setting [10]. RESCUE-Japan [11] randomized 203 patients with low Alberta Stroke Program Early CT Scores (ASPECTS) of 3 to 5 to either MT plus maximal medical management or maximal medical management alone and found a significant increase in the percentage of patients with an mRS score of 0 to 3 at 90 days (31% endovascular-therapy group versus 12.7% medical care group; relative risk [RR] 2.43; 95% confidence interval [CI], 1.35-4.37; *P *= 0.002).

Upcoming and Late-Breaking Trials

ANGEL-ASPECTS, TENSION, and SELECT2 are prospective, open-label, blinded endpoint (PROBE) RCTs in China, the European Union (EU), and an international group comprising North America, the EU, the UK, and New Zealand. The primary objective of these trials is to compare the safety and effectiveness of thrombectomy versus maximal medical management alone in patients with low ASPECTS scores of 3 to 5 (NCT04551664; NCT03094715; NCT03876457). ANGEL-ASPECTS results were published in April 2023 and found that patients with large infarctions (ASPECTS score of 3-5 or core infarct volume of 70-100 cc) who underwent endovascular treatment within 24 hours had better outcomes (generalized odds ratio [OR], 1.37; 95% CI, 1.11-1.69; *P *= 0.004) but more intracerebral hemorrhages than patients randomized to medical management alone [12]. SELECT2 results were also published in April 2023 and showed similar results with better 90-day outcomes in favor of MT in 352 patients with large-volume ischemic strokes (ASPECTS score of 3-5 or core infarct volume >50 cc) randomized 1:1 to endovascular therapy (EVT) within 24 hours or medical management alone (generalized OR 1.51; 95% CI, 1.20-1.89; *P *< 0.001) [13].

Direct to Angiography Suite

ANGIOCAT [14] evaluated a direct transfer to angiography suite protocol for patients with suspected LVO within six hours of symptom onset. The authors found an increased odds of IAT, decreased door-to-reperfusion time (57 vs. 84 minutes), and decreased severity of disability (adjusted OR [AOR] mRS, 2.2; 95% CI, 1.2-4.1; *P *= 0.009).

Stent-Retrievers and/or Aspiration

The Penumbra Separator 3D trial [15] (Penumbra, Alameda, CA) randomized 198 patients with AIS due to LVO within eight hours of symptom onset. Results showed non-inferiority of the Penumbra 3D stent-retriever plus aspiration compared to aspiration alone with similar rates of functional independence (mRS 0-2) at 90 days (45.3% vs. 45.8%; *P *> 0.99), and similar rates of modified thrombolysis in cerebral infarction (mTICI) grade 2b/3 (83.9% vs. 74.4%; 90% CI, −3.6 to 19.6)[13].

The ASTER2 trial [16], a follow-up of the ASTER trial [17], which failed to meet its primary endpoint of angiographic superiority, randomized 408 patients to thrombectomy with contact aspiration plus stent-retriever or stent-retriever alone (CE-marked device chosen by the operator) and found similar rates of eTICI 2c/3 reperfusion (64.5% vs. 57.9%, AOR 1.33, 95% CI, 0.88-1.99) and 90-day functional independence (38% vs. 41.9%; AOR 0.86; 95% CI, 0.56-1.33).

The COMPASS trial [18] randomized 134 patients to a direct aspiration first-pass technique (ADAPT) with the Penumbra aspiration system and 136 patients to a stent-retriever first line (SRFL) technique with Solitaire (Medtronic, Minneapolis, MN) or Trevo (Stryker, Kalamazoo, MI) device (balloon guide and/or aspiration used at the discretion of the treating physician) [19]. The COMPASS trial demonstrated non-­inferior 90-day functional outcomes using ADAPT compared to SRFL (52% vs. 50%; *P*_non-inferiority _= 0.0014) [18].

Upcoming Trials

Retrospective analysis of ADAPT using SOFIA catheters (Microvention, Aliso Viejo, CA) suggested that the device was safe and effective with TICI 2b/3 reperfusion in 86.1%, after first pass in 24.2%, and with rescue stent-retriever in 29.7% of cases [20]. The REal-World Analyses of Stroke-Thrombus Occlusion REtrieval (RESTORE) trial using the SOFIA aspiration system are currently being recruited (NCT04451525).

New Stent-Retrievers

The REDIRECT trial [21] randomized 136 patients with AIS due to LVO within eight hours of symptom onset to evaluate the efficacy of the RECO flow restoration (FR) device (Genesis MedTech, Singapore) compared to the Solitaire stent-retriever. There were similar rates of 90-day functional independence (mRS 0-2), 90-day all-cause mortality, and procedure duration between the two groups [21].

The Tonbridge stent (Tonbridge Medical Technology, Guangzhou, China) was compared to the Solitaire stent-retriever in 208 patients with AIS due to LVO within 6 hours of symptom onset. The Tonbridge device showed non-inferiority, with similar rates of all-cause mortality and 90-day mRS of 0-2 [22].

Prospective Trials

The TIGER trial [23] was a single-arm, multicenter, prospective study of the Tigertriever device (Rapid Medical, Sunrise, FL) involving 160 patients with a National Institutes of Health Stroke Scale (NIHSS) score of ≥8 and AIS due to LVO within eight hours of symptom onset. About 95.7% of patients achieved TICI ≥ 2b reperfusion and 58% of patients had 90-day mRS of 0-2 [23].

Posterior Circulation

The BEST trial aimed to evaluate the efficacy of IAT for basilar occlusion but was terminated early after randomization of 131 patients due to high rates of crossover, poor recruitment, and loss of equipoise [24].

ATTENTION [25] randomized 340 adults with acute basilar artery occlusion (BAO) within 12 hours of symptom onset to EVT or maximal medical management and demonstrated a significantly higher rate of good 90-day functional outcome in the endovascular group (46%) compared to the medical group (23%; *P* < 0.001). 

Similarly, BAOCHE [26] randomized 217 adults with BAO to IAT or maximal medical management within 6 to 24 hours of symptom onset. Functional outcomes were significantly better with IAT (46%) compared to medical management (24%; *P* < 0.001). 

Anesthesia

The General or Local Anesthesia in Intra-Arterial Therapy (GOLIATH) trial [27] was a single-center PROBE trial comparing general anesthesia to conscious sedation in 128 patients undergoing IAT for AIS due to LVO and found similar outcomes between the two groups [27].

Ischemic stroke/thrombectomy and thrombolysis

Thrombectomy With or Without Thrombolysis

Additional trials have focused on the role of intravenous alteplase (IV-tPA or intravenous tissue plasminogen activator) plus IAT compared to IAT alone. The DIRECT-MT trial [28] was a randomized PROBE trial that enrolled 656 eligible patients with AIS within 4.5 hours of LKW. The trial, conducted at 41 academic tertiary care centers in China, demonstrated the non-inferiority of IAT alone compared to a combination of IV-tPA followed by thrombectomy [28].

MR CLEAN NO-IV [29] was an open-label, multicenter, RCT in Europe involving 539 patients who underwent IAT alone or IV-tPA followed by IAT and also demonstrated non-inferiority of IAT alone with similar 90-day outcomes and disability scores [29]. The DEVT [30] trial, conducted at 33 stroke centers in China, also demonstrated non-inferiority of IAT alone compared to IV-tPA plus IAT in 90-day functional independence [30].

The SKIP [31] trial, however, failed to demonstrate non-inferiority of IAT alone compared to IAT following IV thrombolysis among 204 patients with acute LVO. With IAT alone, 59.4% of patients had a favorable functional outcome compared to 57.3% with IV thrombolysis plus IAT (OR, 1.09; 95% CI, 0.63 to ∞), but may have been underpowered [31]. The SWIFT DIRECT [32] trial also failed to demonstrate the non-inferiority of IAT alone compared to IV-tPA plus IAT. A total of 408 patients with anterior circulation LVO within 4.5 hours of symptom onset and NIHSS ≥ 5 were randomized, and thrombectomy alone had decreased rates of TICI 2b/3 reperfusion compared to IV-tPA followed by IAT (91% vs. 96%; *P *= 0.047) [32].

Other Trials

The CHOICE [33] trial was a phase 2b randomized, placebo-controlled, double-blind trial of intraarterial alteplase compared to placebo in 121 patients after IAT, which demonstrated a greater likelihood of excellent 90-day neurological outcome (mRS 0-1) in the treatment arm compared to placebo (59% vs. 40.4%; *P *= 0.047) [33].

Upcoming Trials

DIRECT-SAFE [34] is an international multicenter PROBE non-inferiority trial that aims to randomize 780 patients presenting within 4.5 hours of LKW to either direct IAT or IV-tPA followed by IAT (NCT03494920).

Ischemic stroke/thrombolysis

Time Window and Wake-Up Strokes

ECASS-4 [35] enrolled 119 patients with LVO stroke between 4.5 and 9 hours after symptom onset to either IV-tPA or placebo. The trial was stopped early due to slow recruitment and failed to show a significant benefit over placebo during the extended time window [35].

The WAKE-UP [36] trial compared IV-tPA to placebo in patients with unknown symptom onset. Patients with diffusion restriction on MRI but no corresponding finding on FLAIR were randomized. Alteplase demonstrated significantly improved functional outcomes but higher rates of ICH compared to placebo [36]. 

The EXTEND [37] trial had a similar aim of MRI-guided thrombolysis for LVO stroke patients presenting with an unknown LKW time but was stopped early after the WAKE-UP [36] trial was published in 2018. In patients with a mismatch between DWI and FLAIR on MRI, IV-tPA demonstrated a significant improvement in functional outcome resulting in higher rates of any intracranial hemorrhage (ICH) event at 90 days compared to placebo [37]. 

Similarly, THAWS [38] also aimed to evaluate MRI-guided thrombolysis with IV-tPA in patients with unknown LKW but was stopped early when the WAKE-UP [36] trial was published. Early results failed to show any significant benefit of alteplase over placebo but demonstrated comparable safety [38]. 

Tenecteplase

NOR-TEST 2 [39] was a non-inferiority phase 3 PROBE trial following the NOR-TEST trial [40], which showed 0.4 mg/kg of tenecteplase was similar to 0.9 mg/kg of IV alteplase [40]. NOR-TEST 2 was stopped early due to worse safety and functional outcomes with 0.4 mg/kg tenecteplase compared to the standard alteplase dose. NOR-TEST 2 Part B, with an IV tenecteplase dose of 0.25 mg/kg, is ongoing (NCT01949948).

The AcT trial [41] was a multicenter, open-label, parallel-group, registry-linked RCT conducted across 22 comprehensive stroke centers in Canada. The trial compared 0.25 mg/kg intravenous (IV) tenecteplase to 0.9 mg/kg IV alteplase in patients with LVO presenting within 4.5 hours of symptom onset. IV tenecteplase at 0.25 mg/kg resulted in comparable rates of excellent functional outcome (mRS 0-1) at 90-120 days (36.9% vs. 34.8%). There were similar rates of symptomatic ICH within 24 hours in the tenecteplase (3.4%) and alteplase (3.2%) groups, as well as similar rates of 90-day all-cause mortality (15.3% versus 15.4% respectively) [41].

The original EXTEND-IA TNK trial [42] was a phase 2 multicenter PROBE trial demonstrating that 0.25 mg/kg IV tenecteplase (maximum dose 25 mg) improved reperfusion in patients with LVO within 4.5 hours of symptom onset compared to standard dose IV alteplase [42].

EXTEND-IA TNK Part 2 [43] compared IV tenecteplase at 0.40 to 0.25 mg/kg in patients with LVO due to AIS within 4.5 hours of LKW who subsequently underwent endovascular thrombectomy and demonstrated similar rates of all-cause mortality and symptomatic ICH within 36 hours [43]. Thus, a higher dose of IV tenecteplase did not confer an advantage in patients in whom IAT was planned [43].

Upcoming Trials

TEMPO-2 is an RCT of tenecteplase versus standard of care for minor ischemic stroke or transient ischemic attack (TIA) within 12 hours of onset that is currently recruiting (NCT02398656).

TASTE is an ongoing multicenter PROBE trial comparing 0.25 mg/kg IV tenecteplase to the standard dose of alteplase for patients with large vessel occlusion (LVO) stroke within 4.5 hours of symptom onset (ACTRN12613000243718).

Alternative Thrombolytics

The FRIDA [44] trial was an open-label, non-inferiority RCT conducted at 18 sites in Russia that enrolled 385 patients and compared nonimmunogenic staphylokinase to standard alteplase within 4.5 hours of stroke onset. Staphylokinase was non-inferior to alteplase with similar rates of serious adverse events [44].

Ischemic stroke/TIA

Several trials have investigated the safety and efficacy of dual antiplatelet therapy with clopidogrel or ticagrelor plus aspirin compared to aspirin alone for secondary prevention of stroke following TIA or minor ischemic stroke.

Dual Antiplatelet Therapy

The POINT [45] trial randomized 4,881 patients with minor ischemic stroke or high-risk TIA to either clopidogrel plus aspirin or aspirin alone and was halted early due to a significantly decreased risk of stroke in the dual antiplatelet group at 90 days [45]. However, there was a higher risk of major hemorrhage at 90 days in the dual antiplatelet group (0.9%) compared to aspirin alone (0.4%; hazard ratio, 2.32; 95% CI, 1.10-4.87; *P *= 0.02) [45]. 

The THALES [46] trial randomized 11,016 patients with minor ischemic stroke or TIA who did not undergo thrombectomy to either ticagrelor plus aspirin or aspirin alone. Ticagrelor added to aspirin was superior to aspirin alone in preventing recurrent stroke [46] as well as disabling stroke and death at 30 days [47].

The TARDIS [48] trial compared triple antiplatelet therapy (aspirin, clopidogrel, and dipyridamole) with either clopidogrel alone or combined aspirin and dipyridamole in patients with ischemic stroke or TIA within 48 hours of onset. They found no difference in the rate or severity of recurrent TIAs or strokes but did find a significant increase in the risk of major bleeding among the triple antiplatelet group [48].

CHANCE-2 [49] was a double-blind, placebo-controlled RCT that compared ticagrelor plus aspirin to clopidogrel plus aspirin in carriers of the CYP2C19 loss-of-function mutation that presented with minor ischemic stroke or TIA. Results demonstrated a decreased risk of stroke at 90 days with ticagrelor compared to clopidogrel in patients with the CYP2C19 loss-of-function mutation [49].

Updates

The CHANCE [50] trial, first published in 2013, demonstrated the superiority of aspirin plus clopidogrel over aspirin alone for the prevention of recurrent stroke in patients with TIA or minor ischemic stroke treated within 24 hours. A 2018 subgroup analysis found a 50% risk reduction in stroke recurrence in patients with multiple acute infarctions treated with both aspirin and clopidogrel compared with aspirin alone - a finding not seen in patients with one or fewer acute infarctions [51].

Minor Nondisabling Strokes

Among 313 patients with minor non-disabling strokes (NIHSS 0-5), the PRISMS study [52] concluded that alteplase or aspirin had no significant effect on 90-day functional outcomes. However, the study was terminated early precluding any definitive conclusions [52].

Ischemic stroke/blood pressure

Blood Pressure Goals

The ENCHANTED [53] trial randomized 2,227 patients within six hours of AIS onset to either intensive systolic blood pressure (SBP) management (SBP 130-140 mmHg) or standard therapy (SBP < 180 mmHg) for 72 hours. Although intensive BP management was safe with fewer intracerebral hemorrhage events than the intensive group (14.8% vs. 18.7%; *P *= 0.0137), there was no difference in 90-day functional status. The CHASE [54] trial also failed to demonstrate a significant benefit with a 10% to 15% SBP reduction compared to standard SBP in 90-day rates of dependence or death [54].

BP-TARGET [55] did not find a significant reduction in the rate of intraparenchymal hemorrhage or hypotensive events after intensive SBP lowering (SBP 100-129 mmHg) compared to a standard SBP target (SBP 130-185 mmHg) after successful IAT in patients with LVO stroke [55].

Upcoming Trials

INDIVIDUATE [56] is a single-center PROBE trial that randomized 250 patients with LVO undergoing IAT to either a standard intraprocedural SBP goal (SBP 140-180 mmHg) or an individualized approach of SBP maintained at the level on presentation (±10 mmHg).

Ischemic stroke/prehospital

Mobile Stroke Units

Several studies have investigated prehospital interventions for stroke management, including mobile stroke units (MSUs) for triage and intervention. RACECAT [57] randomized 1,401 patients with suspected LVO stroke to either a thrombectomy-capable center or the closest local stroke center and found no significant difference in 90-day neurological outcomes [57]. The Mobile Stroke Unit (MSU) in Rural Areas [57] trial, however, was able to demonstrate that MSUs are valuable for enabling accurate triage decisions for patients with stroke-like symptoms [58]. The RIGHT-2 [59] trial evaluated the safety and efficacy of prehospital administration of transdermal glyceryl trinitrate (GTN) but did not find any improvement in functional outcomes in patients with presumed stroke [59].

Ischemic stroke/other

Statins

There was no difference in clinical outcomes at 90 days in 65 patients with AIS randomized to high-dose or low-dose simvastatin within 24 hours of symptom onset [60].

Glucose Control

The SHINE [61] trial randomized 1,151 patients with AIS within 12 hours of symptom onset to intensive blood glucose control (80-130 mg/dL) or standard glucose control (80-179 mg/dL) for up to 72 hours with no significant difference in favorable functional outcome at 90 days. Hypoglycemia or other adverse events occurred in 11.2% of patients in the intensive arm compared to only 3.2% of patients in the standard treatment arm, and the trial was terminated early as a result [61].

Sonothrombolysis

CLOTBUST-ER [62] randomized 335 patients to sonothrombolysis for patients with AIS due to LVO who were treated with alteplase compared to 341 patients in the control group and found no significant clinical benefit at 90 days [62].

Sphenopalatine Ganglion Stimulation

The ImpACT-24A [63] and ImpACT-24B [64] trials enrolled patients with AIS due to LVO who were not eligible for IAT to sphenopalatine ganglion stimulation 8 to 24 hours after symptom onset. No significant difference in outcomes was found. Subgroup analysis suggested a trend toward improved functional outcomes in patients with radiographic evidence of cortical involvement at presentation.

Ischemia Reperfusion

In the ESCAPE-NA1 [65] trial, IV nerinetide, a neuroprotectant, administered following IAT did not improve functional outcomes at 90 days. Secondary outcomes, including functional and neurological measures of disability, were also similar between groups [65].

Aneurysms

Management

An interim analysis of the ISAT-2 [66] trial, which was a randomized trial of endovascular versus surgical management of ruptured intracranial aneurysms, found complete aneurysm obliteration in 85% (23/27) of the surgical patients compared to 67% (18/27) of the endovascular coiling patients at one-year follow-up, but a higher rate of hospital stays exceeding 20 days in the surgical group (47%) compared to the endovascular group (19%) [66].

Updates

An intent-to-treat analysis of the BRAT [67] trial showed similar mRS scores at any follow-up time for surgical clipping or endovascular coiling but significantly lower rates of retreatment in the surgical group [68]. Ten-year outcomes of the BRAT trial demonstrated better obliteration rates in the surgical group but similar long-term outcomes between groups [69]. A subgroup analysis found better clinical outcomes in posterior circulation aneurysms treated endovascularly at one year, but no difference beyond one year [69].

Upcoming Trials

MCAAT [70] is a multicenter, parallel-group, prospective RCT of the ruptured and unruptured middle cerebral artery (MCA) aneurysms randomized to surgical or endovascular treatment (NCT05161377).

Seizure Prophylaxis

The SPAR [71] trial found no reduction in the rate of early seizures after seven days of perioperative seizure prophylaxis with levetiracetam in patients undergoing surgical treatment of unruptured intracranial aneurysms compared to those who did not receive levetiracetam [71].

New Devices

The PARAT [72] trial investigated the safety and efficacy of the Tubridge (MicroPort NeuroTech, Shanghai, China) flow diverter (FD) compared to Enterprise (Codman, Raynham, MA) stent-assisted coiling for unruptured large/giant intracranial aneurysms [72]. The investigators found a significantly higher rate of obliteration at six months with the Tubridge FD (75.3% vs. 24.5%; OR, 9.4; 95% CI, 4.14-21.38; *P *< 0.001) [72].

The GREAT [73] trial investigated the efficacy of second-generation hydrogel coils compared to bare metal coils and found a significant reduction in aneurysm recurrence, retreatment, morbidity, and death during treatment and follow-up [73]. The HEAT [74] trial similarly found a decreased rate of aneurysm recurrence in patients with small-to-medium aneurysms treated with the second-generation HydroCoil Embolic System (HES; MicroVention, Inc., Aliso Viejo, CA) compared to bare platinum coils [74].

Prospective Cohort Studies

The WEB-IT [75] trial found that the WEB device (MicroVention, Inc.) was safe and effective for wide-neck bifurcation aneurysms [75]. In the TARGET [76] trial of TARGET-360° or helical coils (Penumbra) more than two-thirds of aneurysms achieved long-term complete occlusion [76]. The SCENT [77] trial evaluated the Surpass FD (Stryker Neurovascular, Portage, MI). The CERUS [78] trial investigated the Contour Neurovascular System (Cerus Endovascular, Fremont, CA), and the FRED [79] trial investigated the safety and efficacy of the Flow Redirection Endoluminal Device (MicroVention, Inc.) in the treatment of intracranial aneurysms.

Updates

A five-year update of outcomes from the MAPS [80] trial demonstrated that Matrix2 coils (Boston Scientific, Natick, MA) were non-inferior to bare metal coils but with no significant difference in radiographic or clinical outcomes [81].

A three-year analysis of the WEBCAST and WEBCAST-2 registries found a high safety profile of WEB with adequate occlusion (complete occlusion or neck remnant) in 83.6% of cases [82].

Endovascular Coiling

The DELTA [83] trial demonstrated that 15-caliber coils significantly improved the packing density in 4 to 12 mm unruptured aneurysms compared with 10-caliber coils but had no significant impact on radiographic or clinical outcomes at one year [83].

Prospective Cohort Registry

Long-term results of a post-market, prospective, multicenter registry of the Penumbra SMART COIL system (Penumbra) demonstrated Raymond Roy occlusion I or II in 90.0% of aneurysms and a 6.8% re-treatment rate at one year [84].

Anesthesia

The Deep NMB [85] trial demonstrated improved angiographic image quality during endovascular coiling of unruptured cerebral aneurysm in the group randomized to deep neuromuscular blockade (NMB) compared to the moderate NMB group [85].

Subarachnoid hemorrhage

Aneurysm Re-bleeding

In the ULTRA [86] trial, tranexamic acid (TXA) did not improve clinical outcomes at six months in aneurysmal SAH (aSAH) patients. While no significant difference in the re-bleeding rate was appreciated, there was a favorable trend toward decreased re-bleeding in the TXA group [86].

Upcoming Trials

FIVHeMA [87] is an upcoming trial investigating the safety and efficacy of intraventricular fibrinolysis in aSAH.

Cerebral Vasospasm and Delayed Cerebral Ischemia

Atorvastatin reduced the rate of cerebral vasospasm and infarction in ruptured aneurysms with SAH but did not improve six-month clinical outcomes [88]. Similar findings were seen with pitavastatin, which reduced the rate of radiographic vasospasm compared to placebo (14.8% vs. 33.3%; OR, 0.32; 95% CI, 0.11-0.87; *P *= 0.042) but did not significantly reduce the rate of delayed cerebral ischemia (DCI) or new neurologic deficits [89]. 

IV magnesium sulfate infusion plus oral nimodipine reduced the incidence of DCI and new neurological deficits but did not decrease the incidence of re-hemorrhage or death [90]. A separate study of IV hydrogen therapy plus intracisternal magnesium sulfate infusion in poor-grade SAH patients undergoing surgery similarly demonstrated a reduced incidence of vasospasm and ischemia [91].

In the HIMALAIA [92] trial, patients with aSAH and clinical signs or symptoms of DCI were randomized to induced hypertension or no intervention, but the trial was stopped early due to a lack of effect on cerebral perfusion and slow recruitment. The adjusted risk ratio for poor outcome was 1.0 (95% CI, 0.6-1.8) and the risk ratio for serious adverse events (SAEs) was 2.1 (95% CI, 0.9-5.0), suggesting no significant benefit and an increased risk of SAEs in the treatment group [92].

The PiSAH trial [93] randomized 108 patients to a control group or goal-directed hemodynamic therapy (GDHT) to optimize mean arterial pressure, cardiac index, global end-diastolic index, and extravascular lung water index using vasopressor, inotropes, and crystalloid with specific goals in the presence or absence of vasospasm [93]. Results showed GDHT reduced the rate of DCI after aSAH from 32% in the control group to 13% in the GDHT group (OR, 0.32; 95% CI, 0.11-0.86; *P *= 0.021), with improved functional outcome three months after discharge.

Intracerebral hemorrhage

Tranexamic Acid

The TXA for hyperacute primary Intracerebral Hemorrhage (TICH-2) [94] trial randomized 2,325 participants to 1 g of IV-TXA followed by an eight-hour infusion or a matching placebo administered within eight hours of symptom onset. The trial found a reduction in early deaths at seven days but no significant difference in functional status at 90 days [94].

Hematoma Evacuation

MISTIE III [95] was an international, multicenter, phase 3 PROBE trial that included 499 adult patients with spontaneous supratentorial ICH of 30 cc or greater and compared minimally invasive catheter evacuation followed by thrombolysis to standard medical management. The study group found that ICH evacuation was safe but not effective at improving functional outcomes for one year [95].

Fluoxetine

The FMRICH [96] trial found that fluoxetine initiated within 10 days of symptomatic ICH and maintained for three months was safe and effective at increasing motor recovery at 90 days [96].

Upcoming Trials

Hematoma evacuation: The Endoscopic IVH [97] trial is a multicenter, prospective, RCT in China that will randomize 956 patients with moderate-to-severe intraventricular hemorrhage (IVH) to either endoscopic evacuation or external ventricular drainage, with a primary endpoint of survival at 12 months (NCT04037267).

Critical care/anesthesia: The ASSICHH [98] trial is a multicenter, prospective, RCT in China that aims to enroll 354 subjects in early, rapid blood pressure stabilization with either analgesic (remifentanil and dexmedetomidine) or antihypertensive (urapidil, nicardipine, and labetalol) medications. 

Subdural hematoma

Dexamethasone

In an interim analysis of the first registered prospective randomized placebo-controlled trial (PRPCT) of adjuvant dexamethasone [99], 47 patients who underwent evacuation and drainage for chronic subdural hematoma (cSDH) were randomized to either a two-week dexamethasone taper or placebo [99]. There were fewer recurrences in the dexamethasone group (0/23, 0%) compared to the placebo group (5/24, 20.83%; *P *= 0.049) and no significant difference in morbidity, mortality, or length of stay [99].

The Dex-CSDH [100] trial, however, found fewer favorable outcomes and more adverse events in the dexamethasone group compared to placebo, including hyperglycemia, new-onset diabetes, new-onset psychosis, and infections [100]. The trial found fewer repeat surgeries for recurrent SDH in the dexamethasone group (6/349 patients, 1.7%) compared to the placebo group (25/350, 7.1%) [100].

Prednisone

The HEMACORT [101] trial found that postoperative prednisone administered at a dose of 1 mg/kg/day followed by weekly stepwise tapering of 10 mg/day demonstrated an earlier radiographic resolution but led to increased rates of SAEs including sleep disorders. 

Atorvastatin

In the ATOCH [102] trial, patients with cSDH were treated nonsurgically and randomized to either 20 mg of atorvastatin or placebo daily for eight weeks. Results showed a 12.5 mL greater reduction in hematoma volume and fewer surgeries for clinical deterioration in the atorvastatin group (11/98, 11.2%) compared to the placebo group (23/98, 23.5%; hazard ratio, 0.47; 95% CI, 0.24-0.92; *P *= 0.03) [102].

Subdural Drains

In the cSDH-Drain trial [103], subperiosteal drains (SPDs) were non-inferior to subdural drains (SDDs) after the burr-hole evacuation of cSDH, although there was a trend toward lower recurrence, fewer surgical infections, and fewer drain misplacements with SPDs [103].

Anesthesia

The inhalational anesthesia [104] trial demonstrated that total IV-propofol infusion provided better brain relaxation, lower intracranial pressure, and better hemodynamics to inhalational anesthesia with sevoflurane in patients with acute subdural hematoma undergoing emergency evacuation [104].

Hypothermia

In patients with acute subdural hematomas requiring emergent evacuation, the HOPES [105] trial found no statistically significant difference in functional outcome between patients randomized to a core temperature of 35 °C before dura opening followed by 33 °C for 48 hours compared with normothermia of 37 °C [105].

Cerebral venous thrombosis

Endovascular Therapy

The TO-ACT [106] trial was a multicenter, international RCT that enrolled 67 patients with symptomatic or deep CVT to either EVT or standard medical management. The trial failed to show any significant improvement in functional outcomes in the EVT group. There was no significant difference in morbidity or mortality between the two groups. Due to a small sample size, future trials should investigate the role of EVT in symptomatic or deep CVT [106].

Anticoagulation

The RE-SPECT CVT [107,108] trial randomized 120 patients with CVT to dabigatran or warfarin for 24 weeks. There were no recurrent VTEs, a low rate of ICH (0% and 3.3%, respectively), and similar rates of recanalization (60.0% and 67.3%, respectively) [107,108].

Arteriovenous malformations

ARUBA Final Follow-Up

A final follow-up of the ARUBA trial published in the Lancet in 2020 [109] found that multimodal treatment of selected patients with brain AVMs did better than the ARUBA intervention arm and similar to the ARUBA medical arm at five years, suggesting that the controversial results of the original ARUBA trial [110,111] ­­remain in question.

Carotid artery atherosclerosis

Anesthesia

The SedLine [112] trial evaluated processed electroencephalogram (EEG)-guided anesthesia management in patients undergoing carotid endarterectomy (CEA) and found a reduced risk of postoperative delirium in these patients.

In the SONOBIRDIE [113] trial, 210 patients were randomized 1:1 to CEA with local anesthesia (LA) or general anesthesia (GA). The study authors found an increased rate of clinically silent radiographic strokes in the GA group compared to the LA group but no difference in clinical outcomes or other complications [113].

Vertebral artery atherosclerosis

Endovascular Stenting

The Vertebral Artery Ischemia Stenting RCT (VIST RCT) [114] was a PROBE clinical trial comparing vertebral artery angioplasty and stenting to best medical management in 182 patients with symptomatic vertebral artery stenosis of at least 50% or more. The trial found no difference in risk of the primary endpoint between the two groups but failed to meet its target recruitment and suffered from a high rate of unconfirmed stenosis in the stented group [114]. 

Extracranial-intracranial bypass

Anesthesia

Only two studies were published on extracranial-intracranial (EC-IC) bypass. The dobutamine versus phenylephrine [115] trial was a randomized crossover study that found both dobutamine and phenylephrine increased graft flow during EC-IC bypass surgery. The sevoflurane and hyperperfusion syndrome [116] study found that sevoflurane post-conditioning did not increase the rate of symptomatic cerebral hyperperfusion (SCH) after EC-IC bypass in patients with Moyamoya disease [116].

Limitations

Our literature review has some limitations. To make our review more feasible, we were only able to include phase 2b or 3 trials and excluded other types of RCTs. Despite efforts to create a comprehensive search strategy, the possibility of excluding pertinent studies remains. The inclusion of our search terms for reproducibility, while not universally converted across all databases, restricts our reporting to the overall number of articles reviewed. It is crucial to acknowledge the enormity of this review, and our findings are current only as of July 1, 2022.

A summary of the major trials is given in Table 1.

**Table 1:** Randomized controlled trials published in the New England Journal of Medicine (NEJM), the Journal of American Medical Association (JAMA), and the Lancet during the study period. TIA, transient ischemic attack

First author	Acronym	Year	Title	Category	Journal
Nogueira et al. [8]	DAWN	2018	Thrombectomy 6 to 24 Hours After Stroke With a Mismatch Between Deficit and Infarct	Ischemic Stroke/Thrombectomy	N Engl J Med
Albers et al. [9]	DEFUSE 3	2018	Thrombectomy for Stroke at 6 to 16 Hours With Selection by Perfusion Imaging	Ischemic Stroke/Thrombectomy	N Engl J Med
Yoshimura et al. [11]	RESCUE-JP	2022	Endovascular Therapy for Acute Stroke With a Large Ischemic Region	Ischemic Stroke/Thrombectomy	N Engl J Med
Requena et al. [14]	ANGIOCAT	2021	Direct to Angiography Suite Without Stopping for Computed Tomography Imaging for Patients With Acute Stroke: A Randomized Clinical Trial	Ischemic Stroke/Thrombectomy	JAMA Neurol
Nogueira et al. [15]	Penumbra Separator 3D	2018	Safety and Efficacy of a 3-Dimensional Stent Retriever With Aspiration-Based Thrombectomy vs Aspiration-Based Thrombectomy Alone in Acute Ischemic Stroke Intervention: A Randomized Clinical Trial	Ischemic Stroke/Thrombectomy	JAMA Neurol
Lapergue et al. [16]	ASTER2	2021	Effect of Thrombectomy With Combined Contact Aspiration and Stent Retriever vs Stent Retriever Alone on Revascularization in Patients With Acute Ischemic Stroke and Large Vessel Occlusion: The ASTER2 Randomized Clinical Trial	Ischemic Stroke/Thrombectomy	JAMA
Turk et al. [18]	COMPASS	2019	Aspiration Thrombectomy Versus Stent Retriever Thrombectomy As First-Line Approach for Large Vessel Occlusion (COMPASS): A Multicentre, Randomised, Open Label, Blinded Outcome, Non-inferiority Trial	Ischemic Stroke/Thrombectomy	Lancet
Liu et al. [24]	BEST	2020	Endovascular Treatment Versus Standard Medical Treatment for Vertebrobasilar Artery Occlusion (Best): An Open-Label, Randomised Controlled Trial	Ischemic Stroke/Thrombectomy	Lancet Neurol
Simonsen et al. [27]	GOLIATH	2018	Effect of General Anesthesia and Conscious Sedation During Endovascular Therapy on Infarct Growth and Clinical Outcomes in Acute Ischemic Stroke a Randomized Clinical Trial	Ischemic Stroke/Thrombectomy	JAMA Neurol
Yang et al. [28]	DIRECT-MT	2020	Endovascular Thrombectomy with or without Intravenous Alteplase in Acute Stroke	Ischemic Stroke/Thrombectomy and Thrombolysis	N Engl J Med
LeCouffe et al. [29]	MR CLEAN-NO	2021	A Randomized Trial of Intravenous Alteplase Before Endovascular Treatment for Stroke	Ischemic Stroke/Thrombectomy and Thrombolysis	N Engl J Med
Zi et al. [30]	DEVT	2021	Effect of Endovascular Treatment Alone vs Intravenous Alteplase Plus Endovascular Treatment on Functional Independence in Patients With Acute Ischemic Stroke: The DEVT Randomized Clinical Trial	Ischemic Stroke/Thrombectomy and Thrombolysis	JAMA
Suzuki et al. [31]	SKIP	2021	Effect of Mechanical Thrombectomy Without vs With Intravenous Thrombolysis on Functional Outcome Among Patients With Acute Ischemic Stroke: The SKIP Randomized Clinical Trial	Ischemic Stroke/Thrombectomy and Thrombolysis	JAMA
Fischer et al. [32]	SWIFT DIRECT	2022	Thrombectomy Alone Versus Intravenous Alteplase Plus Thrombectomy in Patients With Stroke: An Open-Label, Blinded-Outcome, Randomised Non-inferiority Trial	Ischemic Stroke/Thrombectomy and Thrombolysis	Lancet
Renú et al. [33]	CHOICE	2022	Effect of Intra-arterial Alteplase vs Placebo Following Successful Thrombectomy on Functional Outcomes in Patients With Large Vessel Occlusion Acute Ischemic Stroke: The CHOICE Randomized Clinical Trial	Ischemic Stroke/Thrombectomy and Thrombolysis	JAMA
Thomalla et al. [36]	WAKE-UP	2018	MRI-Guided Thrombolysis for Stroke With Unknown Time of Onset	Ischemic Stroke/Thrombolysis	N Engl J Med
Ma et al. [37]	EXTEND	2019	Thrombolysis Guided by Perfusion Imaging Up to 9 Hours After Onset of Stroke	Ischemic Stroke/Thrombolysis	N Engl J Med
Kvistad et al. [39]	NOR-TEST 2, Part A	2022	Tenecteplase Versus Alteplase for the Management of Acute Ischaemic Stroke in Norway (NOR-TEST 2, Part a): A Phase 3, Randomised, Open-Label, Blinded Endpoint, Non-inferiority Trial	Ischemic Stroke/Thrombolysis	Lancet Neurol
Menon et al. [41]	AcT	2022	Intravenous Tenecteplase Compared With Alteplase for Acute Ischaemic Stroke in Canada (AcT): A Pragmatic, Multicentre, Open-Label, Registry-Linked, Randomised, Controlled, Non-inferiority Trial	Ischemic Stroke/Thrombolysis	Lancet
Campbell et al. [43]	EXTEND-IA TNK (Part 2)	2020	Effect of Intravenous Tenecteplase Dose on Cerebral Reperfusion Before Thrombectomy in Patients With Large Vessel Occlusion Ischemic Stroke: The EXTEND-IA TNK Part 2 Randomized Clinical Trial	Ischemic Stroke/Thrombolysis	JAMA
Gusev et al. [44]	FRIDA	2021	Non-immunogenic Recombinant Staphylokinase Versus Alteplase for Patients With Acute Ischaemic Stroke 4·5 H After Symptom Onset in Russia (FRIDA): A Randomised, Open Label, Multicentre, Parallel-Group, Non-inferiority Trial	Ischemic Stroke/Thrombolysis	Lancet Neurol
Johnston et al. [45]	POINT	2018	Clopidogrel and Aspirin in Acute Ischemic Stroke and High-Risk TIA	Ischemic Stroke/TIA	N Engl J Med
Johnston et al. [46]	THALES	2020	Ticagrelor and Aspirin or Aspirin Alone in Acute Ischemic Stroke or TIA	Ischemic Stroke/TIA	N Engl J Med
Amarenco et al. [47]	THALES	2021	Ticagrelor Added to Aspirin in Acute Ischemic Stroke or Transient Ischemic Attack in Prevention of Disabling Stroke: A Randomised Clinical Trial	Ischemic Stroke/TIA	JAMA Neurol
Bath et al. [48]	TARDIS	2018	Antiplatelet Therapy With Aspirin, Clopidogrel, and Dipyridamole Versus Clopidogrel Alone or Aspirin and Dipyridamole in Patients With Acute Cerebral Ischaemia (TARDIS): A Randomised, Open-Label, Phase 3 Superiority Trial	Ischemic Stroke/TIA	Lancet
Wang et al. [49]	CHANCE-2	2021	Ticagrelor Versus Clopidogrel in CYP2C19 Loss-of-Function Carriers With Stroke or TIA	Ischemic Stroke/TIA	N Engl J Med
Khatri et al. [52]	PRISMS	2018	Effect of Alteplase vs Aspirin on Functional Outcome for Patients With Acute Ischemic Stroke and Minor Nondisabling Neurologic Deficits: The PRISMS Randomized Clinical Trial	Ischemic Stroke/Other	JAMA
Anderson et al. [53]	ENCHANTED	2019	Intensive Blood Pressure Reduction With Intravenous Thrombolysis Therapy for Acute Ischaemic Stroke (ENCHANTED): An International, Randomised, Open-Label, Blinded-Endpoint, Phase 3 Trial	Ischemic Stroke/Blood Pressure	Lancet
Mazighi et al. [55]	BP-TARGET	2021	Safety and Efficacy of Intensive Blood Pressure Lowering After Successful Endovascular Therapy in Acute Ischaemic Stroke (BP-TARGET): A Multicentre, Open-Label, Randomised Controlled Trial	Ischemic Stroke/Blood Pressure	Lancet Neurol
Pérez de la Ossa et al. [57]	RACECAT	2022	Effect of Direct Transportation to Thrombectomy-Capable Center vs Local Stroke Center on Neurological Outcomes in Patients With Suspected Large-Vessel Occlusion Stroke in Nonurban Areas: The RACECAT Randomized Clinical Trial	Ischemic Stroke/Pre-Hospital	JAMA
Helwig et al. [58]	Mobile Stroke Unit in Rural Areas	2019	Prehospital Stroke Management Optimized by Use of Clinical Scoring vs Mobile Stroke Unit for Triage of Patients With Stroke: A Randomized Clinical Trial	Ischemic Stroke/Pre-Hospital	JAMA Neurol
Bath et al. [59]	RIGHT-2	2019	Prehospital Transdermal Glyceryl Trinitrate in Patients With Ultra-Acute Presumed Stroke (RIGHT-2): An Ambulance-Based, Randomised, Sham-Controlled, Blinded, Phase 3 Trial	Ischemic Stroke/Pre-Hospital	Lancet
Johnston et al. [61]	SHINE	2019	Intensive vs Standard Treatment of Hyperglycemia and Functional Outcome in Patients With Acute Ischemic Stroke: The SHINE Randomized Clinical Trial	Ischemic Stroke/Other	JAMA
Alexandrov et al. [62]	CLOTBUST-ER	2019	Safety and Efficacy of Sonothrombolysis for Acute Ischaemic Stroke: A Multicentre, Double-Blind, Phase 3, Randomised Controlled Trial	Ischemic Stroke/Other	Lancet Neurol
Bornstein et al. [64]	ImpACT-24B	2019	An Injectable Implant to Stimulate the Sphenopalatine Ganglion for Treatment of Acute Ischaemic Stroke Up to 24 H From Onset (ImpACT- 24B): An International, Randomised, Double-Blind, Sham-Controlled, Pivotal Trial	Ischemic Stroke/Other	Lancet
Hill et al. [65]	ESCAPE-NA1	2020	Efficacy and Safety of Nerinetide for the Treatment of Acute Ischaemic Stroke (ESCAPE-NA1): A Multicentre, Double-Blind, Randomised Controlled Trial	Ischemic Stroke/Other	Lancet
Post et al. [86]	ULTRA	2021	Ultra-Early Tranexamic Acid After Subarachnoid Haemorrhage (ULTRA): A Randomised Controlled Trial	Subarachnoid Hemorrhage	Lancet
Sprigg et al. [94]	TICH-2	2018	Tranexamic Acid for Hyperacute Primary IntraCerebral Haemorrhage (TICH-2): An International Randomised, Placebo-Controlled, Phase 3 Superiority Trial	Intracerebral Hemorrhage	Lancet
Hanley et al. [95]	MISTIE-III	2019	Efficacy and Safety of Minimally Invasive Surgery With Thrombolysis in Intracerebral Haemorrhage Evacuation (MISTIE III): A Randomised, Controlled, Open-Label, Blinded Endpoint Phase 3 Trial	Intracerebral Hemorrhage	Lancet
Hutchinson et al. [100]	Dex-CSDH	2020	Trial of Dexamethasone for Chronic Subdural Hematoma	Subdural Hematoma	N Engl J Med
Jiang et al. [102]	ATOCH	2018	Safety and Efficacy of Atorvastatin for Chronic Subdural Hematoma in Chinese Patients: A Randomized Clinical Trial	Subdural Hematoma	JAMA Neurol
Coutinho et al. [106]	TO-ACT	2020	Effect of Endovascular Treatment With Medical Management vs Standard Care on Severe Cerebral Venous Thrombosis: The TO-ACT Randomized Clinical Trial	Cerebral Venous Thrombosis	JAMA Neurol

## Conclusions

Numerous RCTs were published in cerebrovascular neurosurgery between 2018 and July 2022. RCTs on the management of ischemic stroke, including mechanical thrombectomy and thrombolysis, accounted for the majority of publications. Mechanical thrombectomy within 24 hours was more effective than medical management alone in the DAWN, DEFUSE, and RESILIENT trials. The RESCUE-Japan trial expanded indications for mechanical thrombectomy in patients with low ASPECTS. The demonstrated non-inferiority of IAT alone compared to IV alteplase followed by IAT in the DIRECT-MT, MR CLEAN NO-IV, and DEVT trials directly influenced clinical care. Several trials, including AcT, EXTEND-IA TNK, and EXTEND-IA TNK Part 2, showed non-inferiority of 0.25 mg/kg of IV tenecteplase compared to IV alteplase for LVO stroke within 4.5 hours of symptom onset.

Routine seizure prophylaxis did not improve clinical outcomes for unruptured intracranial aneurysms undergoing surgical intervention and TXA for subarachnoid hemorrhage showed promising but not significant results in the ULTRA trial. TXA and clot evacuation were not successful at improving outcomes in intracerebral hemorrhage patients. In subdural hematoma patients, dexamethasone was associated with worse clinical outcomes but fewer recurrences or repeat surgeries. TO-ACT failed to demonstrate the efficacy of endovascular therapy for CVT, and RE-SPECT CVT showed that both dabigatran and warfarin may be safe and effective treatments for CVT. Although many trials have been performed in stroke, mechanical thrombectomy, and thrombolysis, the remainder of cerebrovascular neurosurgery faces a shortage of RCTs due to numerous limitations. This review helps define the scope of the large RCTs published in the last four years to guide future research and clinical practice.

## References

[1] Huang W, Du R (2019). 2016-2017 clinical trials in cerebrovascular neurosurgery. J Clin Neurosci.

[2] Berkhemer OA, Fransen PS, Beumer D (2015). A randomized trial of intraarterial treatment for acute ischemic stroke. N Engl J Med.

[3] Campbell BC, Mitchell PJ, Kleinig TJ (2015). Endovascular therapy for ischemic stroke with perfusion-imaging selection. N Engl J Med.

[4] Goyal M, Demchuk AM, Menon BK (2015). Randomized assessment of rapid endovascular treatment of ischemic stroke. N Engl J Med.

[5] Jovin TG, Chamorro A, Cobo E (2015). Thrombectomy within 8 hours after symptom onset in ischemic stroke. N Engl J Med.

[6] Saver JL, Goyal M, Bonafe A (2015). Stent-retriever thrombectomy after intravenous t-PA vs. t-PA alone in stroke. N Engl J Med.

[7] Bracard S, Ducrocq X, Mas JL (2016). Mechanical thrombectomy after intravenous alteplase versus alteplase alone after stroke (THRACE): a randomised controlled trial. Lancet Neurol.

[8] Nogueira RG, Jadhav AP, Haussen DC (2018). Thrombectomy 6 to 24 hours after stroke with a mismatch between deficit and infarct. N Engl J Med.

[9] Albers GW, Marks MP, Kemp S (2018). Thrombectomy for stroke at 6 to 16 hours with selection by perfusion imaging. N Engl J Med.

[10] Martins SO, Mont'Alverne F, Rebello LC (2020). Thrombectomy for stroke in the public health care system of Brazil. N Engl J Med.

[11] Yoshimura S, Sakai N, Yamagami H (2022). Endovascular therapy for acute stroke with a large ischemic region. N Engl J Med.

[12] Huo X, Ma G, Tong X (2023). Trial of endovascular therapy for acute ischemic stroke with large infarct. N Engl J Med.

[13] Sarraj A, Hassan AE, Abraham MG (2023). Trial of endovascular thrombectomy for large ischemic strokes. N Engl J Med.

[14] Requena M, Olivé-Gadea M, Muchada M (2021). Direct to angiography suite without stopping for computed tomography imaging for patients with acute stroke: a randomized clinical trial. JAMA Neurol.

[15] Nogueira RG, Frei D, Kirmani JF (2018). Safety and efficacy of a 3-dimensional stent retriever with aspiration-based thrombectomy vs aspiration-based thrombectomy alone in acute ischemic stroke intervention: a randomized clinical trial. JAMA Neurol.

[16] Lapergue B, Blanc R, Costalat V (2021). Effect of thrombectomy with combined contact aspiration and stent retriever vs stent retriever alone on Revascularization in patients with acute ischemic stroke and large vessel occlusion: the Aster2 randomized clinical trial. JAMA.

[17] Lapergue B, Blanc R, Gory B (2017). Effect of endovascular contact aspiration vs stent retriever on Revascularization in patients with acute ischemic stroke and large vessel occlusion: the ASTER randomized clinical trial. JAMA.

[18] Turk AS, Siddiqui A, Fifi JT (2019). Aspiration thrombectomy versus stent retriever thrombectomy as first-line approach for large vessel occlusion (COMPASS): a multicentre, randomised, open label, blinded outcome, non-inferiority trial. Lancet.

[19] Turk AS, Siddiqui AH, Mocco J (2018). A comparison of direct aspiration versus stent retriever as a first approach ('COMPASS'): protocol. J Neurointerv Surg.

[20] Marnat G, Barreau X, Detraz L (2019). First-line Sofia aspiration thrombectomy approach within the endovascular treatment of ischemic stroke multicentric registry: efficacy, safety, and predictive factors of success. AJNR Am J Neuroradiol.

[21] Cao J, Lin H, Lin M (2020). RECO flow restoration device versus solitaire FR with the intention for thrombectomy study (REDIRECT): a prospective randomized controlled trial. J Neurosurg.

[22] Zhang Y, Hua W, Li Z (2021). Efficacy and safety of a novel thrombectomy device in patients with acute ischemic stroke: a randomized controlled trial. Front Neurol.

[23] Gupta R, Saver JL, Levy E (2021). New class of radially adjustable stentrievers for acute ischemic stroke: primary results of the multicenter Tiger trial. Stroke.

[24] Liu X, Dai Q, Ye R (2020). Endovascular treatment versus standard medical treatment for vertebrobasilar artery occlusion (BEST): an open-label, randomised controlled trial. Lancet Neurol.

[25] Tao C, Nogueira RG, Zhu Y (2022). Trial of endovascular treatment of acute basilar-artery occlusion. N Engl J Med.

[26] Jovin TG, Li C, Wu L (2022). Trial of thrombectomy 6 to 24 hours after stroke due to basilar-artery occlusion. N Engl J Med.

[27] Simonsen CZ, Yoo AJ, Sørensen LH, Juul N, Johnsen SP, Andersen G, Rasmussen M (2018). Effect of general anesthesia and conscious sedation during endovascular therapy on infarct growth and clinical outcomes in acute ischemic stroke a randomized clinical trial. JAMA Neurol.

[28] Yang P, Zhang Y, Zhang L (2020). Endovascular thrombectomy with or without intravenous alteplase in acute stroke. N Engl J Med.

[29] LeCouffe NE, Kappelhof M, Treurniet KM (2021). A randomized trial of intravenous alteplase before endovascular treatment for stroke. N Engl J Med.

[30] Zi W, Qiu Z, Li F (2021). Effect of endovascular treatment alone vs intravenous alteplase plus endovascular treatment on functional independence in patients with acute ischemic stroke: the DEVT randomized clinical trial. JAMA.

[31] Suzuki K, Matsumaru Y, Takeuchi M (2021). Effect of mechanical thrombectomy without vs with intravenous thrombolysis on functional outcome among patients with acute ischemic stroke: the SKIP randomized clinical trial. JAMA.

[32] Fischer U, Kaesmacher J, Strbian D (2022). Thrombectomy alone versus intravenous alteplase plus thrombectomy in patients with stroke: an open-label, blinded-outcome, randomised non-inferiority trial. Lancet.

[33] Renú A, Millán M, San Román L (2022). Effect of intra-arterial alteplase vs placebo following successful thrombectomy on functional outcomes in patients with large vessel occlusion acute ischemic stroke: the CHOICE randomized clinical trial. JAMA.

[34] Mitchell PJ, Yan B, Churilov L (2022). Direct-safe: A randomized controlled trial of DIRECT endovascular clot retrieval versus standard bridging therapy. J Stroke.

[35] Ringleb P, Bendszus M, Bluhmki E (2019). Extending the time window for intravenous thrombolysis in acute ischemic stroke using magnetic resonance imaging-based patient selection. Int J Stroke.

[36] Thomalla G, Simonsen CZ, Boutitie F (2018). MRI-guided thrombolysis for stroke with unknown time of onset. N Engl J Med.

[37] Ma H, Campbell BC, Parsons MW (2019). Thrombolysis guided by perfusion imaging up to 9 hours after onset of stroke. N Engl J Med.

[38] Koga M, Yamamoto H, Inoue M (2020). Thrombolysis with alteplase at 0.6 mg/kg for stroke with unknown time of onset: a randomized controlled trial. Stroke.

[39] Kvistad CE, Næss H, Helleberg BH (2022). Tenecteplase versus alteplase for the management of acute ischaemic stroke in Norway (NOR-TEST 2, part A): a phase 3, randomised, open-label, blinded endpoint, non-inferiority trial. Lancet Neurol.

[40] Logallo N, Novotny V, Assmus J (2017). Tenecteplase versus alteplase for management of acute ischaemic stroke (NOR-TEST): a phase 3, randomised, open-label, blinded endpoint trial. Lancet Neurol.

[41] Menon BK, Buck BH, Singh N (2022). Intravenous tenecteplase compared with alteplase for acute ischaemic stroke in Canada (AcT): a pragmatic, multicentre, open-label, registry-linked, randomised, controlled, non-inferiority trial. Lancet.

[42] Campbell BC, Mitchell PJ, Churilov L (2018). Tenecteplase versus alteplase before thrombectomy for ischemic stroke. N Engl J Med.

[43] Campbell BC, Mitchell PJ, Churilov L (2020). Effect of intravenous tenecteplase dose on cerebral reperfusion before thrombectomy in patients with large vessel occlusion ischemic stroke: the EXTEND-IA TNK part 2 randomized clinical trial. JAMA.

[44] Gusev EI, Martynov MY, Nikonov AA (2021). Non-immunogenic recombinant staphylokinase versus alteplase for patients with acute ischaemic stroke 4·5 h after symptom onset in Russia (FRIDA): a randomised, open label, multicentre, parallel-group, non-inferiority trial. Lancet Neurol.

[45] Johnston SC, Easton JD, Farrant M (2018). Clopidogrel and aspirin in acute ischemic stroke and high-risk TIA. N Engl J Med.

[46] Johnston SC, Amarenco P, Denison H (2020). Ticagrelor and aspirin or aspirin alone in acute ischemic stroke or TIA. N Engl J Med.

[47] Amarenco P, Denison H, Evans SR (2020). Ticagrelor added to aspirin in acute ischemic stroke or transient ischemic attack in prevention of disabling stroke: a randomized clinical trial. JAMA Neurol.

[48] Bath PM, Woodhouse LJ, Appleton JP (2018). Antiplatelet therapy with aspirin, clopidogrel, and dipyridamole versus clopidogrel alone or aspirin and dipyridamole in patients with acute cerebral ischaemia (TARDIS): a randomised, open-label, phase 3 superiority trial. Lancet.

[49] Wang Y, Meng X, Wang A (2021). Ticagrelor versus clopidogrel in CYP2C19 loss-of-function carriers with stroke or TIA. N Engl J Med.

[50] Wang Y, Wang Y, Zhao X (2013). Clopidogrel with aspirin in acute minor stroke or transient ischemic attack. N Engl J Med.

[51] Jing J, Meng X, Zhao X (2018). Dual antiplatelet therapy in transient ischemic attack and minor stroke with different infarction patterns: subgroup analysis of the CHANCE randomized clinical trial. JAMA Neurol.

[52] Khatri P, Kleindorfer DO, Devlin T (2018). Effect of alteplase vs aspirin on functional outcome for patients with acute ischemic stroke and minor nondisabling neurologic deficits: the PRISMS randomized clinical trial. JAMA.

[53] Anderson CS, Huang Y, Lindley RI (2019). Intensive blood pressure reduction with intravenous thrombolysis therapy for acute ischaemic stroke (ENCHANTED): an international, randomised, open-label, blinded-endpoint, phase 3 trial. Lancet.

[54] Yuan F, Yang F, Zhao J (2021). Controlling hypertension after severe cerebrovascular event (CHASE): a randomized, multicenter, controlled study. Int J Stroke.

[55] Mazighi M, Richard S, Lapergue B (2021). Safety and efficacy of intensive blood pressure lowering after successful endovascular therapy in acute ischaemic stroke (BP-TARGET): a multicentre, open-label, randomised controlled trial. Lancet Neurol.

[56] Chen M, Kronsteiner D, Möhlenbruch MA (2021). Individualized blood pressure management during endovascular treatment of acute ischemic stroke under procedural sedation (INDIVIDUATE) - an explorative randomized controlled trial. Eur Stroke J.

[57] Pérez de la Ossa N, Abilleira S, Jovin TG (2022). Effect of direct transportation to thrombectomy-capable center vs local stroke center on neurological outcomes in patients with suspected large-vessel occlusion stroke in nonurban areas: the RACECAT randomized clinical trial. JAMA.

[58] Helwig SA, Ragoschke-Schumm A, Schwindling L (2019). Prehospital stroke management optimized by use of clinical scoring vs mobile stroke unit for triage of patients with stroke: a randomized clinical trial. JAMA Neurol.

[59] (2019). Prehospital transdermal glyceryl trinitrate in patients with ultra-acute presumed stroke (RIGHT-2): an ambulance-based, randomised, sham-controlled, blinded, phase 3 trial. Lancet.

[60] Uransilp N, Chaiyawatthanananthn P, Muengtaweepongsa S (2018). Efficacy of high-dose and low-dose simvastatin on vascular oxidative stress and neurological outcomes in patient with acute ischemic stroke: a randomized, double-blind, parallel, controlled trial. Neurol Res Int.

[61] Johnston KC, Bruno A, Pauls Q (2019). Intensive vs standard treatment of hyperglycemia and functional outcome in patients with acute ischemic stroke: the SHINE randomized clinical trial. JAMA.

[62] Alexandrov A V., Köhrmann M, Soinne L (2019). Safety and efficacy of sonothrombolysis for acute ischaemic stroke: a multicentre, double-blind, phase 3, randomised controlled trial. Lancet Neurol.

[63] Bornstein NM, Saver JL, Diener HC (2019). Sphenopalatine ganglion stimulation to augment cerebral blood flow: a randomized, sham-controlled trial. Stroke.

[64] Bornstein NM, Saver JL, Diener HC (2019). An injectable implant to stimulate the sphenopalatine ganglion for treatment of acute ischaemic stroke up to 24 h from onset (ImpACT- 24B): an international, randomised, double-blind, sham-controlled, pivotal trial. Lancet.

[65] Hill MD, Goyal M, Menon BK (2020). Efficacy and safety of nerinetide for the treatment of acute ischaemic stroke (ESCAPE-NA1): a multicentre, double-blind, randomised controlled trial. Lancet.

[66] Darsaut TE, Roy D, Weill A (2019). A randomized trial of endovascular versus surgical management of ruptured intracranial aneurysms: Interim results from ISAT2. Neurochirurgie.

[67] McDougall CG, Spetzler RF, Zabramski JM, Partovi S, Hills NK, Nakaji P, Albuquerque FC (2012). The Barrow ruptured aneurysm trial. J Neurosurg.

[68] Spetzler RF, Zabramski JM, McDougall CG, Albuquerque FC, Hills NK, Wallace RC, Nakaji P (2018). Analysis of saccular aneurysms in the Barrow ruptured aneurysm trial. J Neurosurg.

[69] Spetzler RF, McDougall CG, Zabramski JM (2019). Ten-year analysis of saccular aneurysms in the Barrow ruptured aneurysm trial. J Neurosurg.

[70] Darsaut TE, Keough MB, Boisseau W (2022). Middle cerebral artery aneurysm trial (MCAAT): a randomized care trial comparing surgical and endovascular management of MCA aneurysm patients. World Neurosurg.

[71] Daou BJ, Maher CO, Holste K (2020). Seizure prophylaxis in unruptured aneurysm repair: a randomized controlled trial. J Stroke Cerebrovasc Dis.

[72] Liu JM, Zhou Y, Li Y (2018). Parent artery reconstruction for large or giant cerebral aneurysms using the tubridge flow diverter: A multicenter, randomized, controlled clinical trial (PARAT). AJNR Am J Neuroradiol.

[73] Taschner CA, Chapot R, Costalat V (2018). Second-generation hydrogel coils for the endovascular treatment of intracranial aneurysms a randomized controlled trial. Stroke.

[74] Bendok BR, Abi-Aad KR, Ward JD (2020). The hydrogel endovascular aneurysm treatment trial (HEAT): a randomized controlled trial of the second-generation hydrogel coil. Neurosurgery.

[75] Arthur AS, Molyneux A, Coon AL (2019). The safety and effectiveness of the Woven EndoBridge (WEB) system for the treatment of wide-necked bifurcation aneurysms: final 12-month results of the pivotal WEB Intrasaccular Therapy (WEB-IT) Study. J Neurointerv Surg.

[76] Zaidat OO, Castonguay AC, Rai AT (2019). TARGET® intracranial aneurysm coiling prospective multicenter registry: Final analysis of peri-procedural and long-term safety and efficacy results. Front Neurol.

[77] Kan P, Mohanty A, Meyers PM (2023). Treatment of large and giant posterior communicating artery aneurysms with the surpass streamline flow diverter: results from the SCENT trial. J Neurointerv Surg.

[78] Liebig T, Killer-Oberpfalzer M, Gal G (2022). The safety and effectiveness of the contour neurovascular system (contour) for the treatment of bifurcation aneurysms: the CERUS study. Neurosurgery.

[79] McDougall CG, Diaz O, Boulos A (2022). Safety and efficacy results of the Flow Redirection Endoluminal Device (FRED) stent system in the treatment of intracranial aneurysms: US pivotal trial. J Neurointerv Surg.

[80] McDougall CG, Johnston SC, Gholkar A (2014). Bioactive versus bare platinum coils in the treatment of intracranial aneurysms: the MAPS (Matrix and Platinum Science) trial. AJNR Am J Neuroradiol.

[81] McDougall CG, Johnston SC, Hetts SW (2021). Five-year results of randomized bioactive versus bare metal coils in the treatment of intracranial aneurysms: the Matrix and Platinum Science (MAPS) Trial. J Neurointerv Surg.

[82] Pierot L, Szikora I, Barreau X (2023). Aneurysm treatment with the Woven EndoBridge (WEB) device in the combined population of two prospective, multicenter series: 5-year follow-up. J Neurointerv Surg.

[83] Raymond J, Ghostine J, van Adel BA (2020). Does increasing packing density using larger caliber coils improve angiographic results of embolization of intracranial aneurysms at 1 year: a randomized trial. AJNR Am J Neuroradiol.

[84] Spiotta AM, Park MS, Bellon RJ (2021). The smart registry: Long-term results on the utility of the penumbra smart coil system for treatment of intracranial aneurysms and other malformations. Front Neurol.

[85] Kim BY, Chung SH, Park SJ, Han SH, Kwon OK, Chung JY, Kim JH (2020). Deep neuromuscular block improves angiographic image quality during endovascular coiling of unruptured cerebral aneurysm: a randomized clinical trial. J Neurointerv Surg.

[86] Post R, Germans MR, Tjerkstra MA (2021). Ultra-early tranexamic acid after subarachnoid haemorrhage (ULTRA): a randomised controlled trial. Lancet.

[87] Gaberel T, Gakuba C, Fournel F (2019). FIVHeMA: Intraventricular fibrinolysis versus external ventricular drainage alone in aneurysmal subarachnoid hemorrhage: a randomized controlled trial. Neurochirurgie.

[88] Chen J, Li M, Zhu X (2020). Atorvastatin reduces cerebral vasospasm and infarction after aneurysmal subarachnoid hemorrhage in elderly Chinese adults. Aging (Albany NY).

[89] Naraoka M, Matsuda N, Shimamura N (2018). Long-acting statin for aneurysmal subarachnoid hemorrhage: a randomized, double-blind, placebo-controlled trial. J Cereb Blood Flow Metab.

[90] Zhang C, Zhao S, Zang Y (2018). Magnesium sulfate in combination with nimodipine for the treatment of subarachnoid hemorrhage: a randomized controlled clinical study. Neurol Res.

[91] Takeuchi S, Kumagai K, Toyooka T, Otani N, Wada K, Mori K (2021). Intravenous hydrogen therapy with intracisternal magnesium sulfate infusion in severe aneurysmal subarachnoid hemorrhage. Stroke.

[92] Gathier CS, van den Bergh WM, van der Jagt M (2018). Induced hypertension for delayed cerebral ischemia after aneurysmal subarachnoid hemorrhage a randomized clinical trial. Stroke.

[93] Anetsberger A, Gempt J, Blobner M (2020). Impact of goal-directed therapy on delayed ischemia after aneurysmal subarachnoid hemorrhage: randomized controlled trial. Stroke.

[94] Sprigg N, Flaherty K, Appleton JP (2018). Tranexamic acid for hyperacute primary IntraCerebral Haemorrhage (TICH-2): an international randomised, placebo-controlled, phase 3 superiority trial. Lancet.

[95] Hanley DF, Thompson RE, Rosenblum M (2019). Efficacy and safety of minimally invasive surgery with thrombolysis in intracerebral haemorrhage evacuation (MISTIE III): a randomised, controlled, open-label, blinded endpoint phase 3 trial. Lancet.

[96] Marquez-Romero JM, Reyes-Martínez M, Huerta-Franco MR, Ruiz-Franco A, Silos H, Arauz A (2020). Fluoxetine for motor recovery after acute intracerebral hemorrhage, the FMRICH trial. Clin Neurol Neurosurg.

[97] Zhu J, Tang C, Cong Z, Yang J, Cai X, Liu Y, Ma C (2020). Endoscopic intraventricular hematoma evacuation surgery versus external ventricular drainage for the treatment of patients with moderate to severe intraventricular hemorrhage: a multicenter, randomized, controlled trial. Trials.

[98] Dong R, Li F, Xu Y, Chen P, Maegele M, Yang H, Chen W (2018). Safety and efficacy of applying sufficient analgesia combined with a minimal sedation program as an early antihypertensive treatment for spontaneous intracerebral hemorrhage: a randomized controlled trial. Trials.

[99] Mebberson K, Colditz M, Marshman LA, Thomas PA, Mitchell PS, Robertson K (2020). Prospective randomized placebo-controlled double-blind clinical study of adjuvant dexamethasone with surgery for chronic subdural haematoma with post-operative subdural drainage: interim analysis. J Clin Neurosci.

[100] Hutchinson PJ, Edlmann E, Bulters D (2020). Trial of dexamethasone for chronic subdural hematoma. N Engl J Med.

[101] Ng S, Boetto J, Huguet H (2021). Corticosteroids as an adjuvant treatment to surgery in chronic subdural hematomas: a multi-center double-blind randomized placebo-controlled trial. J Neurotrauma.

[102] Jiang R, Zhao S, Wang R (2018). Safety and efficacy of atorvastatin for chronic subdural hematoma in Chinese patients: a randomized clinical trial. JAMA Neurol.

[103] Soleman J, Lutz K, Schaedelin S, Kamenova M, Guzman R, Mariani L, Fandino J (2019). Subperiosteal vs subdural drain after burr-hole drainage of chronic subdural hematoma: a randomized clinical trial (cSDH-drain-trial). Neurosurgery.

[104] Preethi J, Bidkar PU, Cherian A, Dey A, Srinivasan S, Adinarayanan S, Ramesh AS (2021). Comparison of total intravenous anesthesia vs. inhalational anesthesia on brain relaxation, intracranial pressure, and hemodynamics in patients with acute subdural hematoma undergoing emergency craniotomy: a randomized control trial. Eur J Trauma Emerg Surg.

[105] Hergenroeder GW, Yokobori S, Choi HA (2022). Hypothermia for patients requiring evacuation of subdural hematoma: a multicenter randomized clinical trial. Neurocrit Care.

[106] Coutinho JM, Zuurbier SM, Bousser MG (2020). Effect of endovascular treatment with medical management vs standard care on severe cerebral venous thrombosis: the TO-ACT randomized clinical trial. JAMA Neurol.

[107] Ferro JM, Coutinho JM, Dentali F (2019). Safety and efficacy of dabigatran etexilate vs dose-adjusted warfarin in patients with cerebral venous thrombosis: a randomized clinical trial. JAMA Neurol.

[108] Ferro JM, Bendszus M, Jansen O (2022). Recanalization after cerebral venous thrombosis. A randomized controlled trial of the safety and efficacy of dabigatran etexilate versus dose-adjusted warfarin in patients with cerebral venous and dural sinus thrombosis. Int J Stroke.

[109] Mohr J, Overbey J, Hartmann A (2020). Medical management with interventional therapy versus medical management alone for unruptured brain arteriovenous malformations (ARUBA): final follow-up of a multicentre, non-blinded, randomised controlled trial. Lancet Neurol.

[110] Bambakidis NC, Cockroft KM, Hirsch JA, Connolly ES, Amin-Hanjani S, Meyers PM, Friedlander RM (2014). The case against a randomized trial of unruptured brain arteriovenous malformations: misinterpretation of a flawed study. Stroke.

[111] Mohr JP, Parides MK, Stapf C (2014). Medical management with or without interventional therapy for unruptured brain arteriovenous malformations (ARUBA): a multicentre, non-blinded, randomised trial. Lancet.

[112] Xu N, Li LX, Wang TL (2021). Processed multiparameter electroencephalogram-guided general anesthesia management can reduce postoperative delirium following carotid endarterectomy: a randomized clinical trial. Front Neurol.

[113] Orlický M, Hrbáč T, Sameš M (2019). Anesthesia type determines risk of cerebral infarction after carotid endarterectomy. J Vasc Surg.

[114] Markus HS, Larsson SC, Dennis J (2019). Vertebral artery stenting to prevent recurrent stroke in symptomatic vertebral artery stenosis: the VIST RCT. Health Technol Assess.

[115] Akkermans A, van Waes JA, van Doormaal TP (2020). Effects of dobutamine and phenylephrine on cerebral perfusion in patients undergoing cerebral bypass surgery: a randomised crossover trial. Br J Anaesth.

[116] Yoon HK, Oh H, Lee HC (2020). Effect of sevoflurane Postconditioning on the incidence of symptomatic cerebral hyperperfusion after revascularization surgery in adult patients with Moyamoya disease. World Neurosurg.

